# Out-of-Hospital Cardiac Arrest due to Coronary Spasm with Recurrent Ventricular Fibrillation

**DOI:** 10.1155/2020/8823306

**Published:** 2020-09-04

**Authors:** Vrijraj Sinhji Rathod, Tanmay Kanitkar, Grigoris Karamasis

**Affiliations:** Essex Cardiothoracic Centre, Basildon University Hospital, Nethermayne, Basildon, UK SS16 5NL

## Abstract

We present a case of ventricular fibrillation (VF) secondary to ischaemia induced by coronary artery spasm. An 82-year-old man initially presented with an out-of-hospital VF arrest. On return of spontaneous circulation (ROSC), he was found to be in fast atrial fibrillation (AF); an invasive coronary angiogram revealed unobstructed coronary arteries. During his hospital stay, he developed chest pain, with concomitant ST elevation on ECG (electrocardiogram), which spontaneously resolved. A repeat coronary angiography revealed coronary spasm. Later, he had further ST elevation resulting in ventricular fibrillation. It became clear his initial presentation was most likely due to coronary vasospasm rather than a plaque-rupture or ventricular scar-related event, and he was thus successfully treated with multiple vasodilators and an implantable cardiac defibrillator. This case report highlights how conventional imaging modalities may not always lead to a diagnosis.

## 1. Introduction

Coronary artery spasm resulting in arrhythmia is a rare but recognised cause of syncope and sudden cardiac death [1]. However, if the coronary artery spasm does not cause chest pain, the diagnosis and management are very difficult. Diagnosis of the silent coronary artery spasm is possible if the attack occurs under ECG monitoring; however, it may not occur during ambulatory monitoring or during exercise testing. Long-term surveillance such as repeated ambulatory Holter ECGs or an implantable loop recording may be needed to establish the diagnosis. Since calcium channel blockers (CCB) do not eliminate the risk of further events, combination therapy with CCB and an implantable cardioverter defibrillator (ICD) may be appropriate.

## 2. Case Report

**Figure 1:**
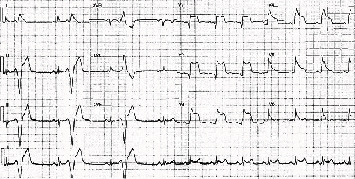
ECG showing ST elevation in the anterior leads while the patient was experiencing chest pain.

**Figure 2:**
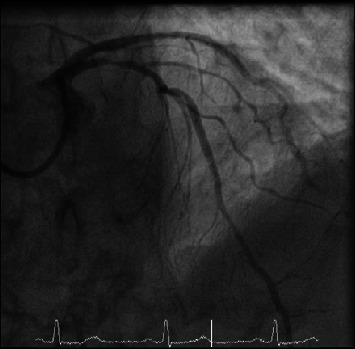
Coronary angiogram illustrating spasm in the LAD.

**Figure 3:**
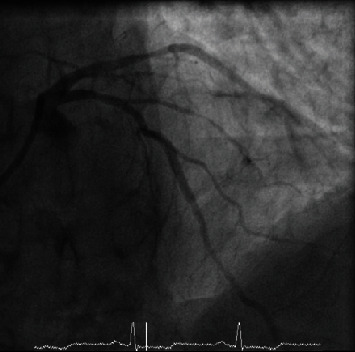
After administration of IV nitrates and resolution of the narrowing in the LAD.

**Figure 4:**
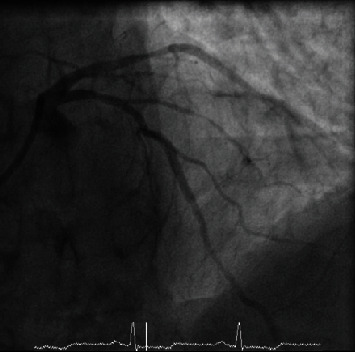
Ventricular fibrillation.

An 82-year-old man presented with an out-of-hospital VF arrest. He reportedly felt very unwell whilst in a supermarket, before suffering from angina and collapsing. He was promptly attended by an ambulance crew who found he was initially in fast AF whilst conscious, before descending into VF, requiring cardiac pulmonary resuscitation (CPR) and subsequent DC cardioversion (DCCV). ROSC was achieved after 10 minutes, where he was found to be in fast AF with anterior ST elevation on ECG, resulting in a HEMS (Helicopter Emergency Medicine Service) transfer for possible primary percutaneous coronary intervention. His cardiovascular risk factors included hypertension, possible previously untreated MI, type 2 diabetes mellitus, previous smoker, and poor compliance with all medication whilst also suffering longstanding chronic obstructive pulmonary disease (COPD). On angiography, he was found to have nonflow limiting moderate plaque disease in the left anterior descending artery (LAD), with no significant atheroma in the other coronary arteries. He was thus returned to the ward, clinically stable and without any coronary intervention. Furthermore, his troponin T results suggested he had not suffered a myocardial infarction, rising from an initial 44 to just 48 units (3 and 6 hours, normal < 14 units), which could be explained by fast AF, CPR, or DCCV—in combination or alone. His resting ECG (electrocardiogram) showed T wave inversion in the anterior leads suggesting previous myocardial insult. His electrolytes were all within range, with a potassium of 4.1 mmol/l, sodium of 137 mmol/l, and magnesium of 0.9 mmol/l. His Creatinine was 100 *μ*mol/l and glucose was 7.1 mmol/l. A cardiac MRI was carried out, which identified a left ventricle scar that can be a plausible explanation for his initial VF arrest, with MI unlikely due to a lack of surrounding oedema and reasonable LV contractility. During his stay in the ward, he had further chest pain and ST elevation (Figure 1) in the anterior leads which self-resolved. On subsequent review of his angiographic images, the consensus was he should have a further coronary angiogram with a pressure wire study to the LAD. During the angiogram, the LAD went into coronary spasm (Figure 2) which was associated with further chest pain and ST elevation, which resolved once IV nitrates were administrated through the coronary catheter (Figure 3). He was clinically stable throughout the procedure and returned to the ward for a provisional plan to implant an ICD. He suffered three further VF arrests that same night, which made clear the diagnosis. Each episode of VF was preceded by both chest pain and significant ST elevation in anterior leads before descending into VF (Figure 4), which was terminated by prompt DCCV. The following morning, an ICD was inserted with concomitant vasodilator therapy started. He received a combination of diltiazem and bisoprolol, which he tolerated well and remained stable on. He was discharged soon after, with no shocks delivered by his ICD and no further episodes of chest pain in the ward.

## 3. Discussion

Cardiac ischaemia secondary to coronary spasm is a well-known pathology, but risk of developing ventricular arrhythmia from the sequela is still rare. Like our patient, who was initially admitted with VF and subsequently had mild coronary artery disease on an angiogram and no structural heart disease on imaging, it can be challenging to establish coronary spasm as the underlying substrate for ventricular arrhythmia. Conventionally, these patients have a low recurrence rate; hence, these patients are given a presumed diagnosis on clinical suspicion or misdiagnosed to a have small thromboembolic phenomenon that leads to the event. Fortunately, this patient had a recurrence of his symptoms during his admission, and we were able to identify the underlying aetiology on a repeat coronary angiogram. Even though this case has been described before, this case gave us a rare opportunity to correlate the clinical features of coronary spasm to angiography findings.

An observation study of patients with ICD implants because of out-of-hospital cardiac arrests secondary to coronary spasm had no events in the 5-year follow-up [2].

Kobayashi et al. have previously published that vasogenic coronary spasm was the precipitant of ventricular arrhythmia in 7% of patients suffering from out hospital cardiac arrests [3]. Matsue et al. followed 23 patients who had out-of-hospital VF/VT arrests and then underwent an ICD implant. These patients had no structural heart disease or obstructive coronary disease but did have coronary spasm with acetylcholine change. After a follow-up of nearly 3 years, 4 patients had experienced a further episode of VF which activated their device and prevented sudden death [4]. Interestingly, the median time to first appropriate shock was 2.9 years.

A Japanese registry consisting of 1429 patients with coronary spasms included 35 patients with VF as a primary manifestation of their coronary spasm, 14 patients had an ICD implant, and 2 patients from the ICD implant group had appropriate shock. They also discovered that patients who had their medication discontinued or reduced were more at risk of sudden cardiac death and nonfatal myocardial infarction [5].

The above data corroborates the risk of further ventricular arrhythmias in this group of patients. Currently, calcium channel blockers and smoking cessation are main forms of therapy for coronary spasm and angina episodes [6]. There is no randomised multicentre trial which has compared the ICD implant with medical therapy versus medical therapy alone. Aoki et al. reported a 0.6% per year rate of cardiac arrest in patients only receiving medical therapy for coronary spasm [7]. The above data constructs an overwhelming case of significant risk of cardiac arrest in this cohort of patients; therefore, a clinical trial consisting of an arm receiving only medical therapy would be deemed unethical. We have retrospective cohort studies which have emphasised the high degree of reoccurrence of ventricular arrhythmia patients with coronary spasm, but these events occur after 12 months of the primary event. Our case is unique because the patient had multiple episodes of spasm within a short period of time and gave us the opportunity of capturing the aetiology on angiography.

## Additional Points

*Learning Points*. (i) Coronary spasm is not a benign pathology, and patients should be assessed for reoccurrence and risk for sudden cardiac death. (ii) Medical therapy plays a vital role in preventing further cardiac arrhythmias, but an ICD should be considered. (iii) Coronary spasm should be considered as a differential diagnosis for VF in individuals with unobstructed coronary arteries.
